# A High-Performance Anti-Noise Algorithm for Arrhythmia Recognition

**DOI:** 10.3390/s24144558

**Published:** 2024-07-14

**Authors:** Jianchao Feng, Yujuan Si, Yu Zhang, Meiqi Sun, Wenke Yang

**Affiliations:** 1 School of Electronic and Information Engineering (SEIE), Zhuhai College of Science and Technology, Zhuhai 519041, China; fengjc22@mails.jlu.edu.cn (J.F.); z_y22@mails.jlu.edu.cn (Y.Z.); yangwk@mails.jlu.edu.cn (W.Y.); 2 College of Communication Engineering, Jilin University, Changchun 130012, China; smq2019@126.com

**Keywords:** arrhythmia detection, convolutional optimization, broad learning system, machine learning

## Abstract

In recent years, the incidence of cardiac arrhythmias has been on the rise because of changes in lifestyle and the aging population. Electrocardiograms (ECGs) are widely used for the automated diagnosis of cardiac arrhythmias. However, existing models possess poor noise robustness and complex structures, limiting their effectiveness. To solve these problems, this paper proposes an arrhythmia recognition system with excellent anti-noise performance: a convolutionally optimized broad learning system (COBLS). In the proposed COBLS method, the signal is convolved with blind source separation using a signal analysis method based on high-order-statistic independent component analysis (ICA). The constructed feature matrix is further feature-extracted and dimensionally reduced using principal component analysis (PCA), which reveals the essence of the signal. The linear feature correlation between the data can be effectively reduced, and redundant attributes can be eliminated to obtain a low-dimensional feature matrix that retains the essential features of the classification model. Then, arrhythmia recognition is realized by combining this matrix with the broad learning system (BLS). Subsequently, the model was evaluated using the MIT-BIH arrhythmia database and the MIT-BIH noise stress test database. The outcomes of the experiments demonstrate exceptional performance, with impressive achievements in terms of the overall accuracy, overall precision, overall sensitivity, and overall F1-score. Specifically, the results indicate outstanding performance, with figures reaching 99.11% for the overall accuracy, 96.95% for the overall precision, 89.71% for the overall sensitivity, and 93.01% for the overall F1-score across all four classification experiments. The model proposed in this paper shows excellent performance, with 24 dB, 18 dB, and 12 dB signal-to-noise ratios.

## 1. Introduction

### 1.1. Background

Cardiovascular diseases (CVDs) are among the most common causes of death globally, with more people dying from cardiovascular diseases each year than from any other cause [1]. Among them, cardiac arrhythmia is one of the most common and significant cardiovascular diseases, referring to a group of conditions where the heart beats too slowly, too quickly, or irregularly [2]. Arrhythmias can occur not only alone but also in conjunction with other cardiovascular diseases. They impose severe physical and psychological burdens on the patient and can lead to sudden death.

Mild cardiac arrhythmias may not present obvious symptoms, but severe malignant arrhythmias can lead to severe consequences, such as palpitations, chest discomfort, dizziness, and fainting [3]. Additionally, high management costs and associated income losses related to cardiovascular diseases contribute to a significant economic burden. According to predictions, the global impact of cardiovascular diseases is expected to escalate significantly by 2035. It is estimated that more than 130 million individuals worldwide will be affected, resulting in a considerable economic burden on society, with projected costs exceeding 1.1 trillion dollars [4].

As a result, the accurate detection of cardiac arrhythmias has attracted considerable attention from biomedical researchers. Electrocardiogram (ECG) devices are currently effective non-invasive tools for monitoring heart health and diagnosing cardiovascular diseases. However, because of the complexity of ECG recordings, manual diagnosis is labor-intensive and prone to error, especially when fatigue sets in. As a result, computer-assisted automatic heartbeat classification has emerged as a key method in arrhythmia analysis. This technology can quickly and accurately detect and classify heartbeats, helping physicians to make the right diagnosis and treatment decisions to prevent deterioration and life-threatening situations.

### 1.2. Related Works

Recently, scholars, both domestic and international, have proposed various methods for the analysis of cardiac arrhythmias. These methods are primarily based on either traditional machine-learning or deep-learning approaches [5]. These methods have greatly improved the efficiency of arrhythmia diagnosis.

Liu et al. [6] introduced a meta-ensemble network based on metric learning, combining meta-learning and metric learning to address the challenges of few-shot learning. In addition, the method involved a meta-testing strategy using a twin-input ensemble network with N-way K-shots, effectively enhancing the model’s robustness. Experimental results demonstrated that this approach achieved the high-precision diagnosis of cardiac arrhythmias. Tuncer et al. [7] employed a method that combines the discrete wavelet transform (DWT) with a novel one-dimensional hexagonal local pattern (1D-HLP) technique to decompose and extract features from electrocardiogram signals. The final electrocardiogram signal features were obtained through feature dimensionality reduction and fusion, followed by recognition and classification using a k-nearest-neighbor (KNN) classifier. Experimental results indicated a 95.0% classification accuracy for 17 cardiac arrhythmia categories. Marinho et al. [8] proposed a multimodal feature fusion approach, integrating various features, such as Fourier, Goertzel, higher-order statistics (HOS), and structure co-occurrence matrices (SCMs). The method was tested with four well-known classifiers, and experimental results demonstrated its outstanding recognition performance.

Although machine learning has shown promising outcomes in cardiac arrhythmia recognition, it often necessitates considerable resources for feature extraction and selection. This results in models that take a long time to train. Conversely, deep-learning techniques have witnessed rapid advancements in recent years, exhibiting exceptional capabilities across diverse domains, including semantic segmentation [9], image classification [10], facial emotion recognition [11], object detection [12], and numerous others.

Deep learning, a robust technique for feature learning, has the capability to automatically extract valuable feature representations from raw data. In contrast to conventional approaches, deep learning offers the distinct advantage of the direct application to classification tasks, eliminating the requirement for supplementary feature engineering. In the classification of cardiac arrhythmias, researchers widely adopt various deep-learning architectures, such as convolutional neural networks (CNNs) and their variants [13], long short-term memory (LSTM) networks [14,15], deep neural networks (DNNs) [16], and residual neural networks [17]. These powerful architectures play a crucial role in cardiac arrhythmia analysis, achieving remarkable results. Through deep learning, we can more accurately identify and classify cardiac arrhythmias, providing clinicians with better decision support tools and further improving the effectiveness of patient treatments.

Lu et al. [18] proposed a deep separable focal loss convolutional neural network (DSC-FL-CNN) for cardiac arrhythmia classification, where focal loss contributes to improving the performance on imbalanced datasets, especially for small-sample arrhythmias. Experimental results demonstrated an overall macro-average *F*1*-score* of 79% for the model. Islam et al. [19] developed a novel method called HARDC for cardiac arrhythmia classification. It integrates a bidirectional recurrent neural network with mixed hierarchical attention and an expanded convolutional neural network (CNN) to generate fused features. HARDC achieved an *F*1*-score* of 98.21%, an accuracy of 97.66%, and a recall rate of 99.60% for classification. Compared to traditional methods, the expanded CNN with HARDC had a shorter runtime. Hu et al. [20] introduced a transformer-based deep-learning neural network called ECG DETR for continuous single-lead electrocardiogram segment arrhythmia detection. This method can predict both the heartbeat position and category simultaneously without explicit heartbeat segmentation, featuring a compact end-to-end design. Experimental results demonstrated that ECG DETR achieved high accuracy in various arrhythmia detection tasks, with rates of 99.12%, 99.49%, and 99.23% compared to traditional methods. Guo et al. [21] presented the multiscale convolutional causal attention network (MSCANet), which uses a multiscale convolutional neural network in combination with causal convolutional attention mechanisms for ECG signal classification from the PhysioNet MIT-BIH arrhythmia database. At the same time, the dataset is balanced through the downsampling of the majority class and the oversampling of the minority class using SMOTE, effectively categorizing the five heartbeat types in the test dataset.

As deep learning demonstrates outstanding performance, it also faces challenges, such as high computational resource consumption and complex model structures. To address these issues, researchers, including Chen et al. [22], have proposed a novel network structure called the broad learning system (BLS). Compared to deep structures, the BLS is simpler, has fewer parameters, and exhibits higher scalability with lower computational costs. Utilizing ridge regression to compute the network’s connection weights, the BLS avoids common pitfalls in deep learning, such as learning rates, stopping criteria, local minima, and learning cycle issues. Additionally, the BLS can rapidly adjust its network structure through incremental learning algorithms without the need to retrain the entire process. This innovation provides a new perspective for addressing the computational resource demands and complexity issues associated with deep learning.

In recent years, the BLS has gained widespread attention because of its excellent performance and efficient learning efficiency. It has been improved and applied in various fields, including fault detection [23] and control theory [24] among others. Ye et al. [25] proposed a novel deep-learning architecture called DCBLS, which successfully extracts sufficient information from raw data and achieves success in image denoising. The method also features a parallel framework suitable for large-scale data and can adopt adaptive regularization parameter criteria. Experimental results on benchmark datasets show its effectiveness and superiority compared to existing image-denoising methods. Yang et al. [26] introduced the weighted BLS method, which assigns weights to each training sample, with increased weights for minority class samples. They designed a density-based weight generation mechanism to guide the generation of specific weight matrices and proposed the AWBLS model, considering both inter-class and intra-class distances. Finally, they used the IWEB method to enhance the stability and robustness of AWBLS. Guo et al. [27] presented a semi-supervised vehicle-type classification scheme based on the ensemble BLS for intelligent transportation systems (ITSs). By training a set of base BLS classifiers through semi-supervised learning and using a dynamic ensemble structure to obtain the highest type probability, they improved the generalization performance. Experimental results demonstrated the superiority of this method over single BLS classifiers and other approaches.

In the field of ECG processing, Fan et al. [28] have developed a novel learning method, known as the active broad learning system (ABLS), for ECG arrhythmia classification. ABLS has the potential to reduce the time and costs involved in the expert annotation of heartbeats. Furthermore, Fan et al. [29] have extended this work by proposing a class-specific weighted broad learning system (CSWBLS), which is based on the BLS. By constructing a least-square error term by the class and utilizing weights to constrain each class’s contribution to the model, CSWBLS has demonstrated promising outcomes in solving the problem of the category imbalance.

The BLS is known for its simple structure and excellent performance. However, its performance may be less satisfactory when applied to tasks involving the recognition of noisy data.

### 1.3. Proposed Method

To address the mentioned issues, we propose an improved system based on the BLS as a new method for electrocardiogram feature extraction. We adopt a classical cascaded convolutional structure to generate feature nodes for the BLS, replacing the traditional feature node generation process with two cascaded convolutional layers. This modification retains the nonlinear feature-mapping process of the BLS system. The two convolutional layers consist of independent component analysis (ICA) filters and principal component analysis (PCA) filters. Through convolutional operations, these layers enhance the system’s understanding of deep features in the samples. Specifically:

1. ICA is employed to separate mixed signals, thereby obtaining multiple independent signal components. This is particularly useful for processing ECG signals, as they are often composed of signals from multiple physiological sources. Using ICA, the mixed signals can be separated into individual components, allowing for a more accurate analysis and understanding of ECG data;
2. PCA is typically used to eliminate data correlations. In our study, we employ a multi-lead parallel cascaded convolutional structure for the layered learning of arrhythmia features. The convolutional kernels of the two layers are generated by the ICA and PCA algorithms. This cascaded convolutional structure effectively extracts latent and meaningful information when dealing with noisy data;
3. We are using ICA convolution with PCA convolution to enhance the feature extraction capability of the BLS. PCA convolution with ICA convolution is used to replace the shallow-feature-mapping process in the BLS, which, in turn, improves the noise robustness of the model.

### 1.4. Arrangement

The subsequent sections of this paper are structured as follows: Section 2 delves into the details of the databases utilized in the research and outlines the methodology for grouping experimental data. Following that, Section 3 offers a comprehensive exposition of the implementation details of the COBLS. In Section 4, the experimental results are presented, accompanied by a comparative analysis with methodologies found in the existing literature. The conclusive Section 5 encapsulates this study, providing a summary of the research findings and insights obtained throughout the investigation.

## 2. Review of the Broad Learning System

**Figure 1:**
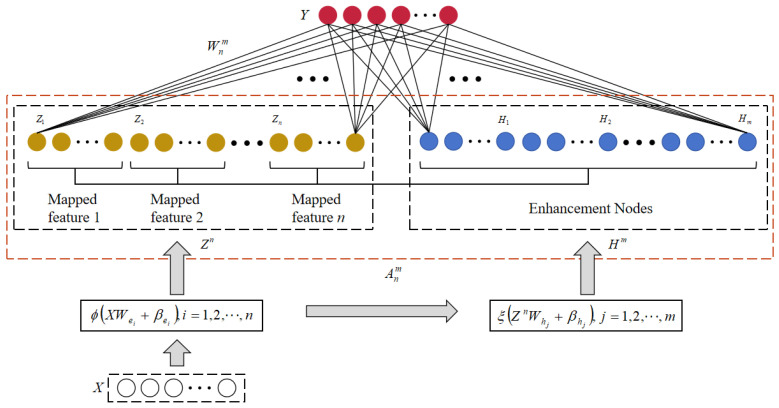
Structure of conventional BLS.

The BLS, introduced by Chen et al. [22], has a flexible neural network structure. Comprising four key components—input, feature nodes, enhancement nodes, and output—the BLS undergoes a series of transformations. Initially, input data are mapped to feature nodes using a simple mapping function. The feature nodes are then enhanced using activation functions, resulting in enhancement nodes. Finally, the feature nodes and enhancement nodes are combined into an expanded matrix, which connects to the output. Notably, the weights and biases between input data and feature nodes, as well as between feature nodes and enhancement nodes, are randomly generated. The association weights between the expanded matrix and the output can be determined using ridge regression theory. See Figure 1.

All the training samples are represented as a matrix denoted by $X\in {ℜ}^{N\times M}$, with the corresponding labels denoted by $Y\in {ℜ}^{N\times L}$. Here, N represents the quantity, M represents the dimensionality of the data in X, and L denotes the number of classes. Assuming that the BLS includes n sets of feature nodes and m sets of enhancement nodes, the feature node ($Z_{i}\in {ℜ}^{N\times k}$) is calculated using Equation (1) as follows:

(1) $$Z_{i}=\varphi \left(XW_{e_{i}}+\beta_{e_{i}}\right),i=1,2,\cdot \cdot \cdot ,n$$

where $W_{e_{i}}\in {ℜ}^{M\times k}$ and $\beta_{e_{i}}\in {ℜ}^{N\times k}$ represent randomly generated weights and biases, respectively. φ(•) is the feature-mapping function. After obtaining all the feature nodes, the enhancement nodes ($H_{j}\in {ℜ}^{N\times p}$) for the *j*th group can be calculated using Equation (2).

(2) $$H_{j}=\xi \left(Z^{n}W_{h_{j}}+\beta_{h_{j}}\right),j=1,2,\cdot \cdot \cdot ,m$$

where $W_{h_{j}}\in {ℜ}^{\left(n\times k\right)\times p}$ and $\beta_{h_{j}}\in {ℜ}^{N\times p}$ are randomly generated weights and biases, respectively. ξ(•) denotes the activation function.

Through the above calculations, we obtain all the feature nodes and enhancement nodes and combine them together to obtain the feature extension matrix ($A_{n}^{m}=[Z^{n}|H^{m}]\in {ℜ}^{N\times \left(n\times k+m\times p\right)}$), where $Z^{n}\equiv \left[Z_{1},Z_{2},\cdot \cdot \cdot ,Z_{n}\right]\in {ℜ}^{N\times \left(n\times k\right)}$ and $H^{m}\equiv \left[H_{1},H_{2},\cdot \cdot \cdot ,H_{m}\right]\in {ℜ}^{N\times \left(m\times p\right)}$. Finally, the feature-expanded matrix ($A_{n}^{m}$) is connected with the output (Y) as follows:

(3) $$Y=A_{n}^{m}W_{n}^{m}$$

Equation (4) demonstrates that the connection weights of the network, represented by $W_{n}^{m}\in {ℜ}^{\left(n\times k+m\times p\right)\times L}$, can be approximated efficiently using the ridge regression of the pseudo inverse of $A_{n}^{m}$, with the actual output (*Y*). This approach can provide accurate estimates of the connection weights, enabling the improved performance of the network.

(4) $$W_{n}^{m}={\left(\lambda I+{\left(A_{n}^{m}\right)}^{T}A_{n}^{m}\right)}^{-1}{\left(A_{n}^{m}\right)}^{T}Y$$

where *I* is the identity matrix. It is worth noting that the Moore–Penrose (MP) inversion of $A_{n}^{m}$ is as follows:

(5) $${\left(A_{n}^{m}\right)}^{+}={\left(\lambda I+{\left(A_{n}^{m}\right)}^{T}A_{n}^{m}\right)}^{-1}{\left(A_{n}^{m}\right)}^{T}$$

Three incremental learning algorithms (enhancement node increment, feature node increment, and input data increment) have been developed within the BLS framework [22]. They allow for the efficient incorporation of new information into the existing model without requiring complete retraining. These algorithms enhance flexibility, scalability, and computational efficiency in the BLS framework.

## 3. Proposed COBLS

This paper employs a convolutional cascade structure to generate feature nodes for the BLS. The feature node generation process in broad learning is replaced by two cascaded convolutional layers, composed of ICA filters and PCA filters. By optimizing the solution process of the feature nodes through PCA convolution and ICA convolution algorithms, this paper delves deeper into extracting data features from ECG signals. The proposed model exhibits a deeper level of understanding capability.

### 3.1. COBLS

This paper proposes an ECG signal recognition model based on the convolutional operator-optimized broad learning algorithm. The model introduces an ICA convolutional operator and a PCA convolutional operator, where the former is based on the ICA algorithm, to extract signal features through convolution, enhancing the model’s capability to handle noisy signals. The latter, utilizing the PCA algorithm, extracts the main components of the target signal. These components are then convolved to reinforce the signal’s features, thus improving the model’s understanding of the underlying depth features.

PCA maps the data from the original high-dimensional space to a low-dimensional space by finding the principal components (i.e., the directions with the highest variance) in the data. Because PCA retains the principal variance of the data, and noise tends to be distributed in dimensions with less variance, PCA removes noise from the data to a certain extent. The features extracted by PCA are linear combinations of the original data, which are better able to reflect the overall structure of the data and the main information.

The goal of the ICA is to separate mixed signals (or data) into statistically independent components. This is in contrast to the linear combination of the PCA, where ICA is able to detect nonlinear relationships in the data and isolate potentially independent source signals. The components extracted by ICA are statistically independent, which means that there is no linear or nonlinear correlation between them.

PCA and ICA are complementary in terms of data degradation and feature extraction: PCA excels at discovering major structures and linear relationships in the data, while ICA is able to further isolate potentially independent source signals. Using a combination of these two methods provides a more comprehensive understanding of the intrinsic structure and information of the data.

**Figure 2:**
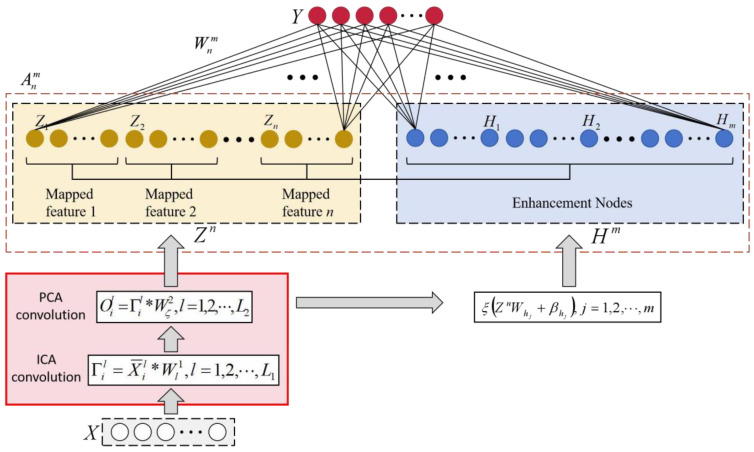
Schematic of the proposed model.

Furthermore, the feature-coding component of the model allows for the re-encoding of the extracted features into vectors. This facilitates the solution of the broad learning model’s problem, enabling efficient and effective analysis and prediction. See Figure 2 and Algorithm 1.
**Algorithm 1: The proposed COBLS****Inputs:** heartbeat matrix X and heartbeat label Y
**Outputs:** network weight $W_{n}^{m}$
1: **for** ICA convolution **do**2:  Calculate the whitening matrix ($V_{L}$) using Equation (8).3:  Calculate the unmixing matrix ($D_{L}$) using the FastICA toolkit.4:  Calculate the ICA convolutional kernel ($W_{l}^{1}$) using Equation (9).5:  The initial eigenmatrix ($\Gamma_{i}^{l}$) is calculated using Equation (10).6: **end for**7:  **for** PCA convolution **do**8:  Construct the second-order pending matrix (U).9:  Calculate the covariance matrix ($UU^{T}$).10:   Extract the co-eigenvector.11:   Calculate the PCA convolutional kernel ($W_{\zeta }^{2}$) using Equation (11).12:   Calculate the second-order eigenmatrix ($O_{i}^{l}$) using Equation (12).13: **end for**14: Calculate the decimal matrix ($T_{i}$) using Equation (13).15: Calculate the histogram feature vector ($f_{i}=\left[Bhist\left(T_{i}^{1}\right),Bhist\left(T_{i}^{2}\right),\cdot \cdot \cdot ,Bhist\left(T_{i}^{p}\right),\right]$).16: Take f as the feature node ($Z^{n}$) of the network.17: **for** j = 1:m **do**18:  Generate random matrices $W_{h_{j}}$ and $\beta_{h_{j}}$.19:  Calculate the enhancement node ($H_{j}$) using Equation (2).20: **end for**21: Calculate the network weight ($W_{n}^{m}$) using Equation (4).

### 3.2. ICA Convolution

The training sample, $X_{N}$, with corresponding labels, $Y_{N}$, are as follows:

(6) $$X=\left[X_{1},X_{2},\cdot \cdot \cdot ,X_{i},\cdot \cdot \cdot ,X_{N}\right]$$

(7) $$Y=\left[Y_{1},Y_{2},\cdot \cdot \cdot ,Y_{i},\cdot \cdot \cdot ,Y_{N}\right]$$

where $X_{i}$ and $Y_{i}$ each belong to the *i*th sample matrix, with its corresponding *i*th label.

Prior to estimating the independent components of the electrocardiogram matrix, a preliminary step is performed by conducting signal component extraction on X. The covariance matrix (C) is computed as $C=XX^{T}$. Then, the eigenvalue matrix (D) and the eigenvector matrix (E) are extracted using $EDE^{T}=C$. The whitening matrix containing the $L_{1}$ column vectors is then obtained using the following equation:

(8) $$V_{L_{1}}=D_{L_{1}}^{-\frac{1}{2}}E_{L_{1}}^{T}$$

where $V_{L_{1}}$ is a whitening matrix.

In this step, the FastICA toolkit is utilized to process sample data for obtaining high-performance ICA filters. The ICA algorithm is employed to obtain the decomposition matrix (W), which estimates the source data (S) based on statistical independence principles. To estimate the orthogonal matrix (*B*), the FastICA toolkit with Gaussian nonlinearity, tanh nonlinearity, and power-3 nonlinearity is employed. Subsequently, the ICA filters are calculated using Equation (9) as follows:

(9) $$W_{l}^{1}=BV_{l},l=1,2,\cdot \cdot \cdot ,L_{1}$$

Then, a set of convolutional operations, as described in Equation (9), is applied to generate the primary feature blocks (PFBs).

(10) $$\Gamma_{i}^{l}={\bar{X}}_{i}^{l}*W_{l}^{1},l=1,2,\cdot \cdot \cdot ,L_{1}$$

where $\Gamma_{i}^{l}$ is the lth feature block of the *i*th ECG matrix (${\bar{X}}_{i}^{l}$), and * denotes convolution. The feature extraction of the first layer of the network can be accomplished by the above operation.

### 3.3. PCA Convolution

In this section, we will describe the PCA convolution performed on the features extracted from the first convolutional layer.

The operation in the PCA convolutional feature extraction stage is similar to that in the ICA convolutional feature extraction stage. First, obtain the PCA convolutional kernels. In this stage, use Equation (11) to obtain the PCA convolutional kernels as follows:

(11) $$W_{\zeta }^{2}=mat_{k_{3},k_{4}}\left(q_{\zeta }\left(UU^{T}\right)\right)\in {ℜ}^{k_{3}\times k_{4}},\zeta \in 1,2,\cdot \cdot \cdot ,L_{2}$$

In this stage, q(•) selects the eigenvectors associated with the $L_{2}$ largest eigenvalues from the covariance matrix ($\Gamma \Gamma^{T}$). Subsequently, these vectors are transformed to $L_{2}$ matrices, which serve as the PCA filters ($W_{\zeta }^{2}$) for further analysis.

According to Equation (12), perform convolution using $\Gamma_{i}^{l}$ and $W_{\zeta }^{2}$, obtaining the output ($O_{i}^{l}$), referred to as the secondary feature block (SFB), as follows:

(12) $$O_{i}^{l}=\Gamma_{i}^{l}*W_{\zeta }^{2}$$

After the above two convolutional operations, we have completed the feature extraction of the original ECG signal.

### 3.4. Feature Coding

To facilitate the processing of the data by the BLS, we encode the extracted features in a vector form. In this section, we describe the use of the Heaviside function to encode the extracted feature blocks into a binary matrix, where positive values equal 1 and other values equal 0. Then, according to Equation (13), we convert this binary matrix to a grayscale image, where the range of each pixel value is $\left[0,2^{L_{1}}-1\right]$.

(13) $$T_{i}=\sum_{l=1}^{L_{1}}2^{l-1}H\left(O_{i}^{l}\right)$$

where $T_{i}$ is the decimal-valued image.

We represent overlapping sample blocks in the decimal matrix. Then, we use a histogram scheme to represent each sample block matrix statistically. Finally, the histogram feature vectors (${\left\{f_{i}=\left[Bhist\left(T_{i}^{j}\right)\right]\right\}}_{j}^{p}$) are combined and concatenated into a comprehensive vector ($f_{i}=\left[Bhist\left(T_{i}^{1}\right),Bhist\left(T_{i}^{2}\right),\cdot \cdot \cdot ,Bhist\left(T_{i}^{p}\right),\right]$), where $T_{i}^{j}$ is the *j*th feature block of the *i*th sample, and p is the sum of these blocks.

By applying the enhancements outlined in Equation (2), we can derive the model’s feature nodes and enhancement nodes, thereby enhancing the output secondary features. Similarly, we only need to solve the model weight matrix problem according to Equation (4). Through equation solving, we can obtain the model weights ($W_{n}^{m}$).

## 4. Experimental Validations

**Figure 3:**
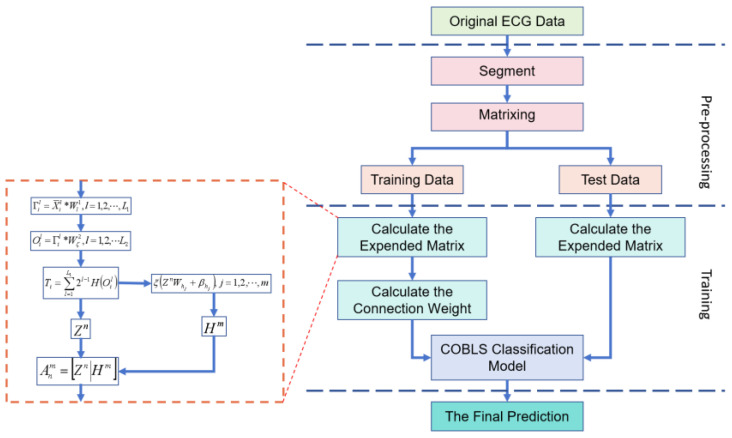
General flowchart of the experimental methodology.

All the experiments were conducted on a workstation equipped with a core (E5-2630V4), CPUs (2.20 GHz × 20), RAM (32.0 GB), and an Nvidia GeForce GTX 1080Ti graphics processor, using the Matlab software platform. Figure 3 illustrates the overall experimental process, which begins by segmenting the ECG signals without denoising. Subsequently, the partitioned data are separated into training and test sets, in which the training set is employed to train the COBLS algorithm, while the test set is used to evaluate the COBLS’s performance.

### 4.1. Materials

In this study, the performance of the COBLS was verified using two commonly used ECG databases downloaded from PhysioNet: the MIT_BIH arrhythmia database [30] and the MIT_BIH noise stress test database [31]. All the data have expert-labeled R-point information and disease labels.

**Figure 4:**
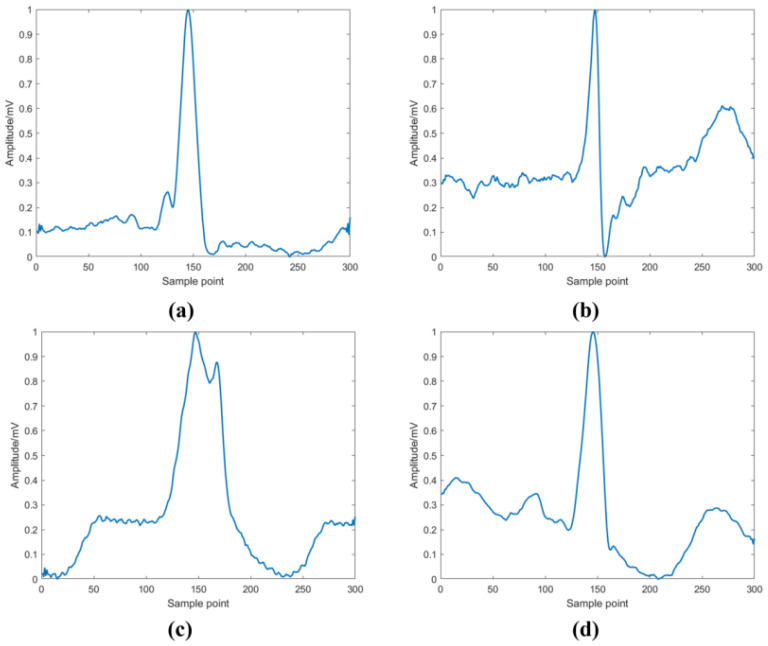
Schematic illustrations of four types of heartbeats: (**a**) N-type heartbeats, (**b**) S-type heartbeats, (**c**) V-type heartbeats, and (**d**) F-type heartbeats.

The four types of beat signals in the experiment are shown in Figure 4, where Figure 4a shows a non-ectopic beat (N), Figure 4b shows a supraventricular ectopic beat (S), Figure 4c shows a ventricular ectopic beat (V), and Figure 4d shows a fusion beat (F).

The experimental data for this study comprised 107,168 ECG segments obtained from 46 labeled recordings containing ML II leads and VI leads. These recordings were a part of the MIT-BIH arrhythmia database, which consists of 48 half-hour excerpts of dual-channel ambulatory ECG recordings. Each signal was sampled at a frequency of 360 Hz, and the database provided R-peaks based reference annotations for each single-cycle signal, serving as foundational information for analysis.

Table 1 shows the various heartbeat types under the five superclasses defined by the AAMI, and this experiment was based on the first four superclasses. Because there are only 15 class Q heartbeats, they are not representative. Therefore, this paper does not include class Q heartbeats in the experiment.

The composition of the data used in the experiment is shown in Table 2, which includes multi-category identification, the identification of each type of disease, and normal (Nb) and abnormal (Ab) identifications. The experiments were conducted using ten-fold cross-validation.

The MIT-BIH noise stress test database was utilized in this study to analyze ECG signals obtained from ML II leads. This database incorporates recordings from two patients sourced from the MIT-BIH arrhythmia database, with varying degrees of noise added to each recording. Specifically, our experimental data comprise ECG recordings with Gaussian white noise added at SNR levels of −6 dB, 0 dB, 6 dB, 12 dB, 18 dB, and 24 dB. For further details regarding the composition of this database, refer to Table 3. The utilization of this database enabled a robust and comprehensive analysis of the performance of our proposed method under various noise conditions.

**Figure 5:**
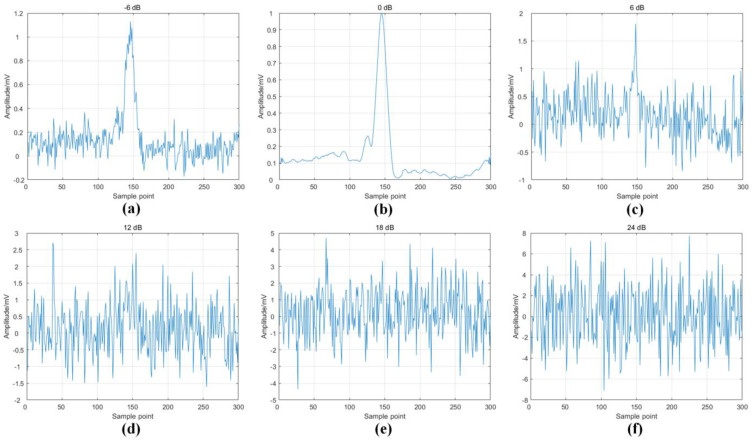
Schematic diagrams of heartbeats for different signal-to-noise ratios.Where, Figure 5 (**a**–**f**) shows the heartbeat schematic for signal-to-noise ratios of -6dB, 0dB, 6dB, 12dB, 18dB, 24dB, respectively.

Figure 5 shows schematics of the heartbeats for six different signal-to-noise ratio cases. We tested the noise–noise robustness of the model on the MIT-BIH noise stress test dataset; the experiment used ten-fold cross-validation, and the data composition is shown in Table 4.

### 4.2. Signal Pre-Processing

To ensure compatibility for subsequent classification phases, it is imperative to segment the ECG signals into individual cyclic signals, commonly referred to as beats. From the MIT-BIH dataset, we extracted 149 sampling points preceding the R-peak and 150 sampling points following the R-peak, based on the annotated R-peak positions in the reference notes. Each sample comprises the entirety of a heartbeat, encompassing 300 sampling points.

**Figure 6:**
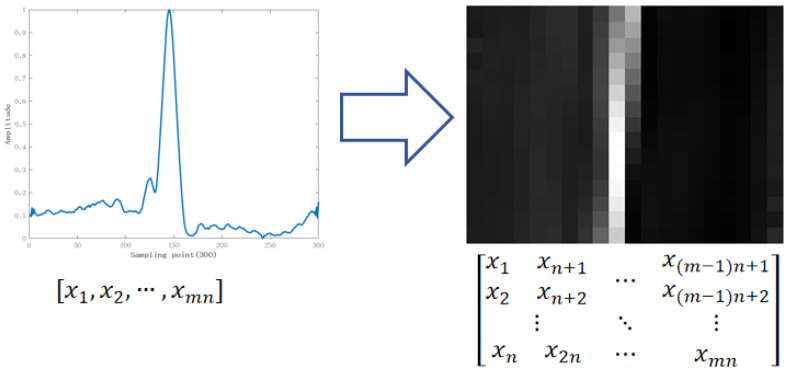
Two-dimensional conversion of a heartbeat signal.

We used a scheme to convert segmented heartbeat signals into a matrix, which enables the two convolutional layers in the model to more fully mine the features of heartbeat signals, effectively improving the feature-mining efficiency of the model. Specifically, the one-dimensional (1D) beat is segmented and arranged into a 2D ECG matrix (pseudo image) with a certain number of rows and columns. The specific processing process is shown in Figure 6 and is based on the transformation equation of the waveform and grayscale image. The one-dimensional data are rearranged into a two-dimensional matrix by the reshape function to increase its sensory field during convolution so that the network can extract more adequate features. In Figure 6, the heartbeat can be viewed as an mn vector with 300 sample points reshaped into an m×n(20×15) matrix. In Figure 6, $x_{i}$ represents the *i*th sampling point of the heartbeat.

Based on each pixel of the sample matrix, block sampling is carried out with a size of $k_{1}\times k_{2}$. Finally, $N\left(a-k_{1}/2\right)\left(b-k_{2}/2\right)$ sample blocks are obtained, and ${\bar{X}}_{i}=\left[{\bar{X}}_{i}^{1},\cdot \cdot \cdot ,{\bar{X}}_{i}^{\lambda },\cdot \cdot \cdot ,{\bar{X}}_{i}^{\left(m-k_{1}/2\right)\left(n-k_{2}/2\right)}\right],i=1,2,\cdot \cdot \cdot ,N$. Among the data, ${\bar{X}}_{i}^{\lambda }$ refers to the vector derived from the *i*th ECG matrix, with a mean value of 0. By performing the aforementioned operations on all the sample data, we can obtain the resulting $X=\left[{\bar{X}}_{1},{\bar{X}}_{2,}\cdot \cdot \cdot ,{\bar{X}}_{N}\right]$.

### 4.3. Evaluation Metrics

In this experimental study, to ensure a fair comparison with previous research, we evaluated our proposed model based on the metrics established in our previous articles [28,29]. These metrics include true positives (TP), true negatives (TN), false positives (FP), and false negatives (FN). Using these four indicators, we calculated the accuracy (Ac), precision ($P_{P}$), sensitivity ($S_{E}$), *F*1*-score*, specificity ($S_{PE}$), average *F*1*-score* (*AF*), average specificity (*ASPE*), and overall accuracy (OA). The *Ac*, $P_{P}$, $S_{E}$, *F*1*-score*, $S_{PE}$, *AF*, *ASPE*, and *OA* are calculated using Equations (14)–(21) as follows:

(14) $$Ac=\frac{TP+TN}{TP+TN+FP+FN}$$

(15) $$P_{P}=\frac{TP}{TP+FP}$$

(16) $$S_{E}=\frac{TP}{TP+FN}$$

(17) $$F1-score=2\times \frac{P_{P}\times S_{E}}{P_{P}+S_{E}}$$

(18) $$S_{PE}=\frac{TN}{TN+FP}$$

(19) $$AF=\frac{\sum_{i=1}^{L}F1-score_{i}}{L}$$

(20) $$ASPE=\frac{\sum_{i=1}^{L}S_{{PE}_{i}}}{L}$$

(21) $$OA=\frac{\sum_{i=1}^{L}TP_{i}}{allECGbeats}$$

where $TP_{i}$ denotes the *TP* of the *i*th class, $F1-score_{i}$ denotes the *F*1*-score* of the ith class, $S_{PE_{i}}$ denotes the recognition accuracy of the *i*th class, and *L* denotes the number of classes.

These metrics allow for a comprehensive assessment of the differences between our proposed model and existing methods, ensuring a rigorous evaluation of its effectiveness and performance.

## 5. Experimental Results and Analysis

### 5.1. Arrhythmia Recognition Experimental Results

The experiments were conducted in accordance with a preset scheme, with Table 1 designed based on AAMI standards. The experimental results were validated using 10-fold cross-validation to ensure the reliability and robustness of our findings.

As presented in Table 5, the overall accuracy rate reached 99.11%, the error rate was 0.89%, and 99.87% of the normal heartbeats were correctly classified. In DS1, the overall accuracy, overall sensitivity, and average *F*1*-score* reached 96.95%, 89.71%, and 93.01%. The sensitivities of class S and class F are only 87.27% and 74.94%, which are lower than those of class N and class V. This poor performance stems from the fact that the training sets of class S and class F contain only 2496 and 722 heartbeat signals, respectively, in each experiment, which are far fewer than those in the training sets of class V and class N. However, considering the four heartbeat types, including class F and class S, the accuracy rate is over 99%.

As shown in Table 6, in DS2, DS3, and DS4, the accuracy rate of the binary classification experiment exceeds 99%. In DS5, all the normal heartbeats and abnormal heartbeats were used for binary classification experiments. Table 7 presents the experimental results, indicating an overall accuracy of 99.01% and an average *F*1*-score* of 97.30%. These results validate the effectiveness of our method in accurately differentiating between ECG records classified as normal or abnormal categories.

### 5.2. Experimental Results of Noise Robustness

The anti-noise performance of the proposed model was evaluated using the MIT-BIH noise stress test database and is summarized in Table 8. Our model achieved the accurate classification of all the beats at signal-to-noise ratios of 24 dB, 18 dB, and 12 dB. However, for signal-to-noise ratios below 12 dB, some heartbeats were misclassified. Notably, across all six noise conditions, the overall classification accuracy exceeded 98%.

As illustrated in Figure 6, the classification accuracy gradually improved with increasing signal-to-noise ratio. This observation suggests that the presence of mixed noise has a detrimental effect on the accurate classification of ECG signals.

Even though the classification performance is adversely affected by high levels of noise, the proposed model demonstrates the effective classification of heartbeat signals when the signal-to-noise ratio exceeds 6 dB. Thus, these findings provide evidence that the proposed model can successfully handle noises in heartbeat signals.

To further validate the superiority of our proposed COBLS model, we conducted comparative experiments with the BLS model using the MIT-BIH noise stress test database. As depicted in Figure 6, our COBLS model outperforms the BLS model, particularly under low signal-to-noise ratio conditions. Remarkably, as the signal-to-noise ratio improves, the recognition accuracy of the COBLS model on the MIT-BIH noise stress test database reaches a remarkable 100%.

### 5.3. COBLS Model Performance Experiments

To test the operational efficiency of the COBLS, we used a CNN, an LSTM, and the BLS and compared their performances with its performance. The CNN model comprises eight layers, utilizes a 1 × 3 convolutional kernel with a stride of 2, and begins with a learning rate of 0.01 over 30 epochs. The LSTM model consists of five layers, with 128 units and a learning rate of 0.01 and spans 10 epochs. In the BLS, the configuration includes 9000 enhancement nodes.

The comparison, as shown in Table 9, reveals that both the COBLS and BLS outperformed the CNN and LSTM in terms of the model training speed and overall efficiency. Although the COBLS lags behind the BLS, the training time required for the COBLS is much shorter compared to those required for traditional deep-learning models.

In this study, the COBLS convolutional parameters for the proposed model were chosen based on reference [32].

**Figure 7:**
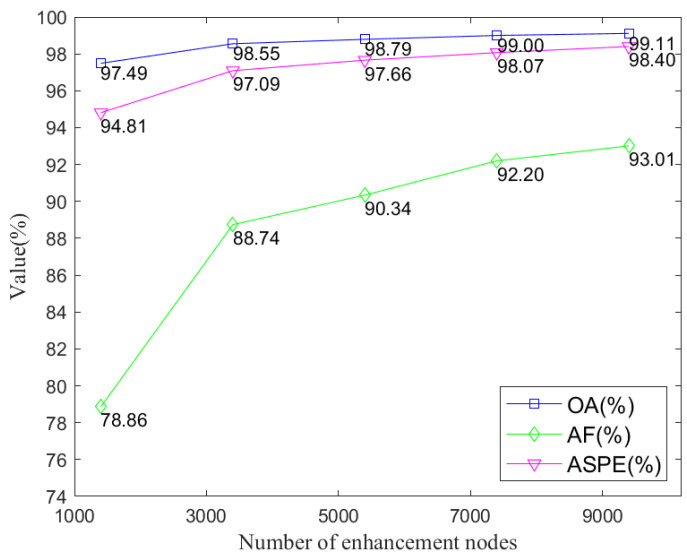
Performance comparison on DS1 for different numbers of enhancement nodes.

To determine the optimal number of feature nodes and reinforcement nodes in the overall broad learning model, we conducted a series of experiments using DS1 as the dataset. The results of these experiments are summarized in Figure 7.

As observed from Figure 7, increasing the number of feature nodes and enhancement nodes in the model leads to gradual improvements in various overall recognition metrics for heartbeats. However, it is worth noting that both the training and testing times of the model also increase proportionally. After multiple rounds of experimental tests, we selected a model configuration with 9000 enhancement nodes. This choice was made by considering the model’s performance across various aspects and weighing the tradeoff between accuracy and computational efficiency.

## 6. Discussion

The early detection of arrhythmias can play a vital role in preventing and mitigating the progression of cardiovascular diseases. The ECG, as a measurement of the heart’s electrical activity, provides critical information about abnormalities in the heart. To overcome the limitations of traditional computer-network-based arrhythmia diagnosis, such as poor recognition of noisy signals and high computational resource requirements, this paper proposes a novel broad learning model with two convolutional layers for ECG signal feature extraction based on the BLS. This approach is specifically designed to effectively deal with noisy ECG signals. The feature vectors extracted from the ECG signals form the feature expansion matrix within the broad learning model, which employs ridge regression theory for data classification. The proposed approach shows significant potential for improving the accuracy and efficiency of arrhythmia diagnoses.

In our study, we used three different schemes (two-class identification, four-class identification, and noise stress tests) to evaluate the effectiveness of the proposed system. The model’s recognition accuracies for two-class (normal and abnormal heartbeats) and four-class (N class, S class, V class, and F class) identifications are 99.01% and 99.55%, respectively. And the overall accuracy rate of the two-class recognition in the test based on the MIT_BIH noise stress test database is higher than 98% under six noise conditions. Moreover, the computational efficiency of the model proposed in this paper is much higher than those of commonly used deep-learning models.

The experimental findings demonstrate the efficacy and universality of the proposed model in accurately classifying ECG signals, even in the presence of noise. This novel approach exhibits a high level of stability and necessitates minimal clinician interaction. As a result, the proposed system holds great potential as a diagnostic tool for analyzing complex data in clinical settings. Its ability to effectively handle noisy signals and its user-friendly nature make it well-suited for practical implementation in healthcare settings, enhancing diagnostic capabilities and facilitating efficient decision-making processes.

All the other researchers listed in Table 10 used the MIT-BIH arrhythmia database as training data. The comparison shows that the COBLS exhibits excellent performance in arrhythmia recognition experiments, and, according to the above theoretical analysis and practical performance, the COBLS is capable for recognizing noise-containing signals. Because the COBLS is based on the improvement of the BLS, the COBLS is not only capable for extracting key features from noisy small-scale signals but also enables the model to be trained at a much higher speed.

## 7. Conclusions

In this paper, we use ICA convolution and PCA convolution for the feature extraction of ECG signals, an operation that replaces the linear feature-mapping process in the BLS. This makes the proposed model perform better in the noise-containing signal recognition task. From the experimental results, it can be seen that the model proposed in this paper has good recognition performance for noisy signals. In addition, our proposed model not only has high recognition accuracy in ECG arrhythmia recognition experiments but also has a high data-processing speed.

In future work, we will focus on further enhancing the real-time and lightweight features of the model so that it can be applicable to wearable devices. This advancement will greatly benefit the timely identification of cardiac arrhythmias and make a significant contribution for saving lives. By prioritizing the development of more efficient and lightweight models, we aim to optimize the performance and applicability of our proposed approach in real-world healthcare settings, ultimately improving patient outcomes and saving lives.

## Figures and Tables

**Table 1:** The AAMI has classified various types of heartbeats into five superclasses.

AAMI Classes	Heartbeat Types
Non-ectopic beats (N)	Normal beat; Left bundle branch block beat; Right bundle branch block beat; Atrial escape beat; Nodal escape beat
Supraventricular ectopic beats (S)	Atrial premature beat; Aberrated atrial premature beat; Nodal premature beat; Supraventricular premature beat
Ventricular ectopic beats (V)	Premature ventricular contraction ventricular escape beat
Fusion beats (F)	Fusion of ventricular and normal beats
Unknown beats (Q)	Paced beat; Fusion of paced and normal beats; Unclassifiable beat

**Table 2:** Experimental dataset.

Dataset	Classes	Class Distribution
DS1	N, S, V, F	89,886; 2773; 6996; 802
DS2	N, S	89,886; 2273
DS3	N, V	89,886; 6996
DS4	N, F	89,886; 802
DS5	Nb, Ab	89,886; 10571

**Table 3:** MIT-BIH noise stress test database details.

Record	SNR (dB)	Record	SNR (dB)
118e24	24	119e24	24
118e18	18	119e18	18
118e12	12	119e12	12
118e06	6	119e06	6
118e00	0	119e00	0
118e_6	−6	119e_6	−6

**Table 4:** Noise robustness experimental data composition.

Dataset	Classes	Class Distribution
DS6	Nb, Ab	3082; 888

**Table 5:** Confusion matrix for all the 10-fold cross-validations of the experimental results in DS1.

Dataset		Predicted				Ac (%)	$P_{P}$ (%)	$S_{E}$ (%)	*F*1-*score* (%)	$S_{PE}$ (%)
	Actual	N	S	V	F					
DS1	N	**89,768**	53	60	5	99.24	99.28	99.87	99.57	93.87
	S	326	**2420**	26	1	99.58	97.38	87.27	92.05	99.93
	V	178	10	**6770**	38	99.63	97.96	96.77	97.36	99.85
	F	144	2	55	**601**	99.76	93.18	74.94	83.07	99.96
*OA* (%)						99.11				

**Table 6:** The confusion matrix of all the 10-fold cross-validation experimental results in DS2, DS3, and DS4.

Dataset		Predicted		Ac (%)	$P_{P}$ (%)	$S_{E}$ (%)	*F*1*-score* (%)	$S_{PE}$ (%)
	Actual	N	S					
DS2	N	**89,819**	67	99.55	99.62	99.93	99.77	87.49
	S	347	**2426**	99.55	97.31	87.49	92.14	99.93
DS3	N	**89,802**	85	99.65	99.72	99.91	99.81	96.37
	V	254	**6743**	99.65	98.76	96.37	97.55	99.91
DS4	N	**89,878**	8	99.78	99.79	99.99	99.89	76.56
	F	188	**614**	99.78	98.71	76.56	86.24	99.99

**Table 7:** Confusion matrix for all the ten-fold cross-validations of the experimental results in DS5.

Dataset		Predicted		Ac (%)	$P_{P}$ (%)	$S_{E}$ (%)	*F*1*-score* (%)	$S_{PE}$
	Actual	Nb	Ab					
DS5	Nb	**89,673**	213	99.01	99.13	99.76	99.45	92.56
	Ab	786	**9785**	99.01	97.87	92.56	95.14	99.76
Average (%)				99.01	98.50	96.16	97.30	96.16
*OA* (%)				99.01				

**Table 8:** COBLS and BLS 10-fold cross-verified results under different signal-to-noise ratios in DS6.

SNR (dB)		COBLS			BLS		
		Predicted			Predicted		
	Actual	Nb	Ab	*OA* (%)	Nb	Ab	*OA* (%)
24	Nb	3082	0		3082	0	
	Ab	0	888	100	0	888	100
18	Nb	3082	0		3082	0	
	Ab	0	888	100	0	888	100
12	Nb	3082	0		3082	2	
	Ab	0	888	100	0	888	
6	Nb	3082	0		3082	6	
	Ab	2	886	99.95	0	886	99.85
0	Nb	3074	8		3074	8	
	Ab	4	884	99.70	10	884	
−6	Nb	3058	24		3058	35	
	Ab	28	860	98.69	27	860	

**Table 9:** Comparison of COBLS, BLS, and LSTM operating speeds in DS6.

	COBLS		BLS		CNN		LSTM	
Noise (dB)	Training Time (s)	Test Time (s)	Training Time (s)	Test Time (s)	Training Time (s)	Test Time (s)	Training Time (s)	Test Time (s)
24	11.03	0.36	4.32	0.08	27.38	0.44	191.22	0.32
18	11.39	0.38	4.30	0.08	27.70	0.46	190.35	0.32
12	11.17	0.35	5.17	0.08	27.54	0.36	190.64	0.32
6	11.09	0.41	4.25	0.07	27.26	0.42	191.01	0.32
0	11.02	0.39	4.40	0.08	27.92	0.46	190.65	0.32
−6	11.20	0.38	4.29	0.08	27.90	0.43	190.21	0.31

**Table 10:** Comparison of the proposed method with other methods in the literature using 10-fold cross-validation on the MIT-BIH database.

Authors	Method	Database	Noise Removal	Class	*OA* (%)/*AF* (%)
Shan et al. [33] (2022)	ECG-AAE	MIT-BIH arrhythmia DB	YES	2(Nb, Ab)	96.73/96.66
Ramkumar, M. et al. [34] (2022)	FFREWT-MLGK-TDCNN	MIT-BIH arrhythmia DB	YES	2(Nb, Ab)	98.00/87.00
Farag et al. [35] (2023)	MF-based CNN	MIT-BIH arrhythmia DB	NO	3(N, S, V)	98.18/92.17
S. Chon et al. [36] (2023)	MKResNet+XForm	MIT-BIH arrhythmia DB	NO	4(N, S, V, F)	97.80/87.90
Zhang et al. [37] (2024)	MRFPN (with L-ROS)	MIT-BIH arrhythmia DB	NO	2(Nb, Ab)	95.04/97.19
Wu et al. [38] (2024)	SC-RGA Transformer	MIT-BIH arrhythmia DB	NO	5(N, S, V, F, Q)	95.70/82.60
This work	COBLS	MIT-BIH arrhythmia DB	NO	2(Nb, Ab)	99.01/97.30
This work	COBLS	MIT-BIH arrhythmia DB	NO	4(N, S, V, F)	99.11/93.01

## Data Availability

All the experiments in this work have been carried out using public open-access ECG databases provided by PhyisoNet, the moniker of the Research Resource for Complex Physiologic Signals, including the MIT-BIH arrhythmia database [30] and MIT-BIH noise stress test database [31], which are cited in this article.
